# Mechanistic Study of Oil Adsorption Behavior and CO_2_ Displacement Mechanism Under Different pH Conditions

**DOI:** 10.3390/molecules30142999

**Published:** 2025-07-17

**Authors:** Xinwang Song, Yang Guo, Yanchang Chen, Shiling Yuan

**Affiliations:** 1 Shandong Key Laboratory of Green Electricity & Hydrogen Science and Technology, School of Chemical Engineering, Shandong Institute of Petroleum and Chemical Technology, Dongying 257061, China; 2022126@sdipct.edu.cn (X.S.); cumt-guoyang@cumt.edu.cn (Y.G.); chenyanchang@sdipct.edu.cn (Y.C.); 2 School of Chemistry and Chemical Engineering, Shandong University, Jinan 250100, China

**Keywords:** oil adsorption behavior, CO_2_ flooding, pH effect, molecular dynamics simulation, quartz surface, interfacial interactions

## Abstract

Enhanced oil recovery (EOR) via CO_2_ flooding is a promising strategy for improving hydrocarbon recovery and carbon sequestration, yet the influence of pH on solid–liquid interfacial interactions in quartz-dominated reservoirs remains poorly understood. This study employs molecular dynamics (MD) simulations to investigate the pH-dependent adsorption behavior of crude oil components on quartz surfaces and its impact on CO_2_ displacement mechanisms. Three quartz surface models with varying ionization degrees (0%, 9%, 18%, corresponding to pH 2–4, 5–7, and 7–9) were constructed to simulate different pH environments. The MD results reveal that aromatic hydrocarbons exhibit significantly stronger adsorption on quartz surfaces at high pH, with their maximum adsorption peak increasing from 398 kg/m^3^ (pH 2–4) to 778 kg/m^3^ (pH 7–9), while their alkane adsorption peaks decrease from 764 kg/m^3^ to 460 kg/m^3^. This pH-dependent behavior is attributed to enhanced cation–π interactions that are facilitated by Na^+^ ion aggregation on negatively charged quartz surfaces at high pH, which form stable tetrahedral configurations with aromatic molecules and surface oxygen ions. During CO_2_ displacement, an adsorption–stripping–displacement mechanism was observed: CO_2_ first forms an adsorption layer on the quartz surface, then penetrates the oil phase to induce the detachment of crude oil components, which are subsequently displaced by pressure. Although high pH enhances the Na^+^-mediated weakening of oil-surface interactions, which leads to a 37% higher diffusion coefficient (8.5 × 10^−5^ cm^2^/s vs. 6.2 × 10^−5^ cm^2^/s at low pH), the tighter packing of aromatic molecules at high pH slows down the displacement rate. This study provides molecular-level insights into pH-regulated adsorption and CO_2_ displacement processes, highlighting the critical role of the surface charge and cation–π interactions in optimizing CO_2_-EOR strategies for quartz-rich reservoirs.

## 1. Introduction

The increasing global demand for energy has led to a growing interest in enhancing the efficiency of hydrocarbon recovery from subsurface reservoirs [1]. Conventional primary and secondary recovery methods often leave a significant fraction of hydrocarbons trapped within the porous rock matrix, which necessitates the development and optimization of tertiary recovery techniques, which are collectively known as enhanced oil recovery (EOR) methods [2,3,4]. Among the various EOR strategies, carbon dioxide (CO_2_) flooding has emerged as a promising approach due to its potential to improve oil mobility, reduce reservoir pressure, and contribute to carbon sequestration efforts [5,6,7]. Despite the technological advancements in CO_2_-EOR, the intricate physicochemical interactions at the solid–liquid interface, particularly in heterogeneous reservoir environments, remain inadequately understood [8,9,10]. Such interactions are critical in determining the ultimate recovery efficiency of this method, especially in formations with considerable mineral compositions such as quartz.

Quartz, as a predominant component in many sandstone reservoirs, provides a chemically active surface that can interact with crude oil components [11,12,13,14]. The adsorption behavior of oil constituents on mineral surfaces can significantly influence oil displacement processes [15,16]. Adsorption not only alters the wettability of the rock but also dictates the extent of oil entrapment and mobilization during EOR operations. Previous experimental and theoretical studies have highlighted that the chemical composition of crude oil, especially its content of polar and aromatic molecules, plays a pivotal role in its interfacial interactions with reservoir minerals [17,18,19,20]. However, the effect of environmental conditions, particularly pH, on these interactions and the subsequent CO_2_ flooding performance remains poorly characterized. Under different pH conditions, the quartz surface undergoes protonation or deprotonation, which leads to variations in the surface hydrophilicity and charge density. At low pH, the protonation of Si-OH groups reduces the surface charge, while at high pH, deprotonation enhances the negative surface charge, potentially strengthening interactions with polar or aromatic oil components [21,22]. These complex interplays suggest that pH could serve as a critical control factor in tailoring the efficiency of CO_2_-driven oil recovery processes. Nevertheless, direct experimental investigations into these phenomena are challenging due to the nanoscale nature of the interactions and the complexity of real reservoir conditions.

Molecular dynamics (MD) simulations offer a powerful computational tool to unravel molecular-level mechanisms that govern adsorption and displacement phenomena at mineral–fluid interfaces. Kirch et al. combined molecular dynamics simulations and machine learning to predict oil–brine interfacial tensions, highlighting the dominant roles of oil properties and salinity [23]. Xue and colleagues used MD simulations to investigate CO_2_–octane displacement and miscibility in calcite nanoslits, revealing strong CO_2_–calcite interactions that enhance oil recovery and CO_2_ sequestration [24]. Lu and colleagues combined microscopic visualization experiments and MD simulations to reveal how hybrid CO_2_ thermal systems decompose heavy oil aggregates, enhance oil recovery, and optimize gas/steam ratios for improved EOR performance [25]. Wang et al. used molecular dynamics simulations with modified combination rules to study the phase behavior and interfacial properties of CO_2_+n–decane systems, revealing that CO_2_-preferential adsorption in quartz nanopores enhances oil displacement and influences molecular diffusion [26]. The motivation for this study stems from the need to elucidate the pH-dependent adsorption behavior of crude oil on quartz surfaces and its impact on CO_2_-EOR efficiency. While experimental studies have provided valuable insights into macroscopic EOR performance, they often lack the resolution to capture molecular-scale phenomena.

**Figure 1:**
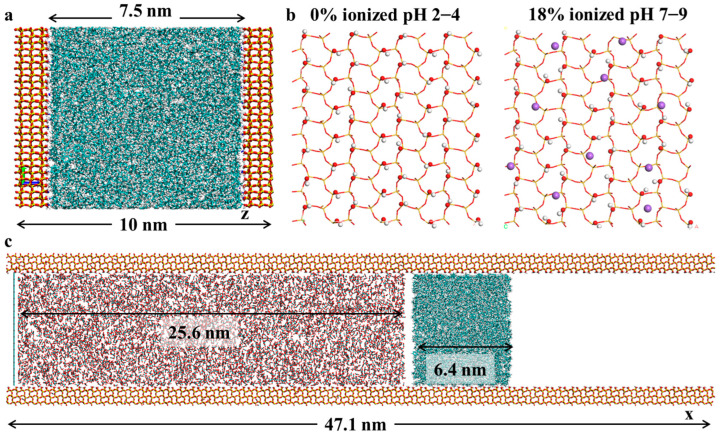
(**a**) Initial model of light crude oil adsorption on the silica surface. (**b**) Silica surface models under different pH conditions. (**c**) Initial structure of the CO_2_ flooding model. Yellow, purple, red, cyan, and white spheres represent Si, Na, O, C, and H atoms, respectively.

In this study, MD simulations were employed to investigate the adsorption behavior of crude oil components on quartz surfaces under different pH environments and to evaluate the influence of these interactions on subsequent CO_2_ displacement processes (Figure 1). Specifically, the distribution of crude oil within silica slits under different environmental conditions, as well as the occurrence states of various crude oil molecules on quartz surfaces, were investigated. Subsequently, the mechanisms of interaction between CO_2_ and crude oil in tight reservoirs and their effects on the miscibility behavior of the CO_2_ were summarized, and the miscibility and displacement mechanisms of CO_2_ molecules were examined in detail. Finally, the kinetic parameters of crude oil components during the displacement process under different pH conditions were evaluated.

## 2. Results and Discussion

### 2.1. Distribution of Crude Oil Within Silica Slits

**Figure 2:**
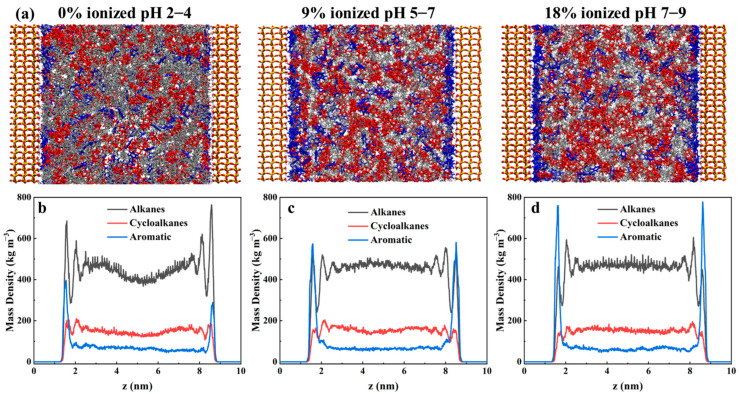
(**a**) Snapshots of the distribution of crude oil molecules within quartz pores under different pH conditions. To distinguish different types of crude oil molecules, alkanes are colored gray, cycloalkanes are colored red, and aromatics are colored blue. Density distributions of various crude oil molecules within the quartz pores under (**b**) pH 2–4, (**c**) pH 5–7, and (**d**) pH 7–9 conditions.

In this section, the distribution states of different types of crude oil molecules within quartz pores under various pH conditions were first investigated. At the start of our molecular dynamics simulations, the hydrocarbon molecules were randomly and homogeneously distributed within the pore space between the quartz surfaces. Figure 2a presents the final equilibrium results after sufficient simulation time. The snapshots from molecular dynamics simulations shown in Figure 2a revealed that alkanes, cycloalkanes, and aromatics were mainly distributed in two regions within the quartz pores: the bulk region and the surface region [27,28]. In the bulk region, these molecules were freely dispersed, exhibiting a high tendency for diffusion. In contrast, near the surface, significant interactions between the molecules and the quartz surface were observed, and these were characterized by stronger adsorption behavior and reduced diffusion tendencies. To further quantify the distribution of crude oil molecules within the quartz slits, the density profiles along the z-direction were calculated, as presented in Figure 2b–d. Overall, the distribution of crude oil molecules in the bulk region of the quartz slit appeared relatively uniform, being primarily concentrated within the range of 2.3–7.4 nm, approximately 1.5 nm away from the quartz surfaces. At the quartz surfaces, pronounced adsorption peaks were observed for all types of crude oil molecules. Specifically, the alkanes formed two distinct adsorption layers at the quartz surface, which indicated relatively strong interactions with the surface. In contrast, the cycloalkanes and aromatics each formed a single, tightly adsorbed layer, which highlighted differences in the surface adsorption behavior among the different molecular types. A comparison of the adsorption behaviors under different pH conditions revealed that the distribution of crude oil molecules in the bulk region remained relatively stable as the pH increased, with the densities of the alkanes, cycloalkanes, and aromatics in the bulk region being maintained at approximately 467 kg/m^3^, 148 kg/m^3^, and 65 kg/m^3^, respectively. However, the adsorption behavior at the quartz surface was significantly affected by the pH. As the pH increased, the adsorption peaks of the alkanes at the quartz surface gradually decreased, while those of the aromatics progressively increased. Specifically, the maximum adsorption peak for the alkanes at the quartz surface decreased from 764 kg/m^3^ to 460 kg/m^3^, whereas the maximum adsorption peak for the aromatics increased from 398 kg/m^3^ to 778 kg/m^3^. These results demonstrate that pH markedly influences the adsorption characteristics of various crude oil components on quartz surfaces.

**Figure 3:**
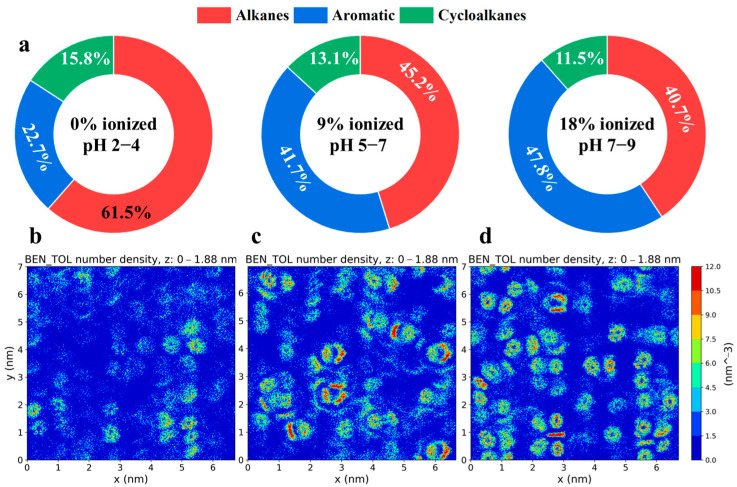
(**a**) Variation in the adsorption proportion of different types of crude oil molecules on the quartz surface as a function of pH. Two-dimensional density distribution maps of aromatic compounds adsorbed on the quartz surface under (**b**) pH 2–4, (**c**) pH 5–7, and (**d**) pH 7–9 conditions.

The surface adsorption behavior of crude oil, which determines its release and flow characteristics in reservoirs, was directly linked to the recoverability and ultimate recovery rate of the crude oil in the reservoir [29,30]. Therefore, the occurrence of different crude oil molecules on quartz surfaces is quantitatively investigated in this section. As shown in Figure 1, three crude oil components (alkanes, cycloalkanes, and aromatic hydrocarbons) were observed to reside on the quartz surface. To further elucidate their behavior on the quartz surface, the contact area proportions of these three components with the quartz surface were calculated under various pH conditions (Figure 3a). It was found through quantitative analysis that the surface contact behavior of these molecules was significantly influenced by different pH environments. A higher contact area with the quartz surface (40.7%) was exhibited by the alkane molecules under low-pH conditions, which was significantly reduced (to 15.8%) under high-pH conditions. A consistent decrease in the surface contact area (from 15.8% to 11.5%) was also observed for the cycloalkane molecules as the pH increased. Conversely, a marked increase in the surface contact area (from 22.7% to 47.8%) was noted for the aromatic hydrocarbon molecules under high-pH conditions, which indicates that stronger interactions with the quartz surface were facilitated in alkaline environments. It was also suggested that aromatic hydrocarbon molecules in the confined oil layers displaced alkane and cycloalkane molecules as the environmental pH increased. This intermolecular substitution effect was found to alter not only the adsorption properties of the quartz surface but also potentially the overall reservoir development strategies. To provide a more intuitive representation of the distribution of aromatic hydrocarbon molecules on the quartz surface, their two-dimensional distribution was calculated and visualized via the *gmx densmap* tool. As depicted in Figure 3b–d, a monolayer adsorption pattern was generally exhibited by the adsorption of aromatic hydrocarbon molecules on the quartz surface. Under low-pH conditions, a relatively sparse distribution and lower adsorption density were observed for the aromatic hydrocarbon molecules on the quartz surface. However, as the pH increased, the surface adsorption density of the aromatic hydrocarbon molecules was found to gradually increase, which indicated a stronger adsorption tendency.

**Figure 4:**
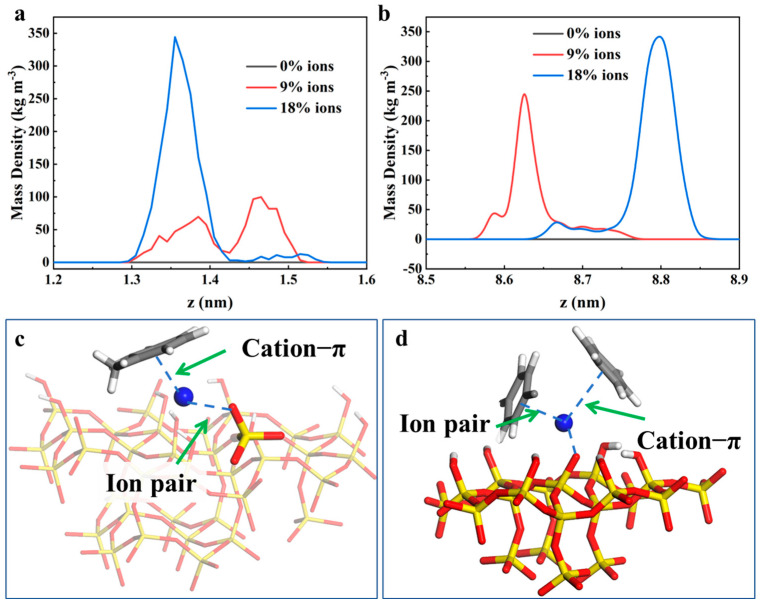
Distribution density of Na^+^ ions near the (**a**) lower and (**b**) upper surfaces of quartz. (**c**,**d**) Adsorption configurations formed by Na^+^ ions and aromatic hydrocarbon molecules on the quartz surface. Yellow denotes silicon (Si) atoms, blue denotes sodium (Na) atoms, red denotes oxygen (O) atoms, and gray denotes carbon (C) atoms.

The reasons for the increased adsorption of aromatic hydrocarbon molecules on quartz surfaces with a rising pH were further investigated. It was considered that the pH environment primarily altered the charge state of the quartz surface, with a higher negative charge typically being exhibited under high-pH conditions [31,32]. On one hand, the π-electron cloud in aromatic hydrocarbon molecules could impart a certain degree of negative charge, which could potentially lead to repulsive forces between these negative charges. However, the adsorption of aromatic hydrocarbon molecules on the quartz surface was still facilitated through mechanisms such as π–π stacking and hydrophobic interactions, owing to their unique structure. On the other hand, an increase in pH was found to potentially modify the ionic strength in the oil layer, allowing Na^+^ ions to aggregate more readily at the surface. This aggregation was hypothesized to enhance the attraction of aromatic hydrocarbon molecules in the oil layer through cation–π interactions. To validate these hypotheses, the density distribution of Na+ ions on the upper and lower quartz surfaces in the oil layer was calculated, as shown in Figure 4a. It was observed that, under pH 5–7 conditions, a relatively broad adsorption peak (approximately 0.2 nm) was formed by the Na^+^ ions on the quartz surface. However, as the pH of the oil layer environment increased to 7–9, the adsorption peak of the Na^+^ ions became narrower (approximately 0.1 nm), with a significantly enhanced peak intensity. This change indicated that the Na^+^ ions were more densely aggregated on the quartz surface under high-pH conditions, which thereby increased the potential for cation–π interactions and significantly enhanced the adsorption density on the quartz surface. To provide a more intuitive representation of this interaction mechanism, the composite structures formed by the Na^+^ ions and aromatic hydrocarbon molecules on the quartz surface were visualized, as shown in Figure 4c,d. Through the repeated examination of interaction snapshots, the composite structures involving Na^+^ ions were broadly categorized into two types. In the first type, as depicted in Figure 4c, the Na^+^ ions were simultaneously engaged in ion–pair interactions with negatively charged oxygen ions in ionized silanol groups on the quartz surface and in cation–π interactions with a nearby aromatic hydrocarbon molecule. In this configuration, the Na^+^ ions acted as an intermediary bridge, promoting the adsorption of aromatic hydrocarbon molecules on the quartz surface. In the second type, as shown in Figure 4d, the Na^+^ maintained ion–pair interactions with negatively charged oxygen ions in ionized silanol groups on the quartz surface while attracting two surrounding aromatic hydrocarbon molecules. A stable tetrahedral interaction configuration was formed through the synergistic ion–pair and cation–π interactions among the quartz surface, Na^+^ ions, and aromatic hydrocarbon molecules, which enabled the stable adsorption of aromatic hydrocarbon molecules on the quartz surface, with a reduced likelihood of dissociation. In summary, the enhanced enrichment of the aromatic hydrocarbon molecules on the quartz surface under high-pH conditions was attributed to the aggregation effect of Na^+^ ions and the strengthened cation–π interactions.

### 2.2. CO_2_ Displacement Process Under Different pH Conditions

**Figure 5:**
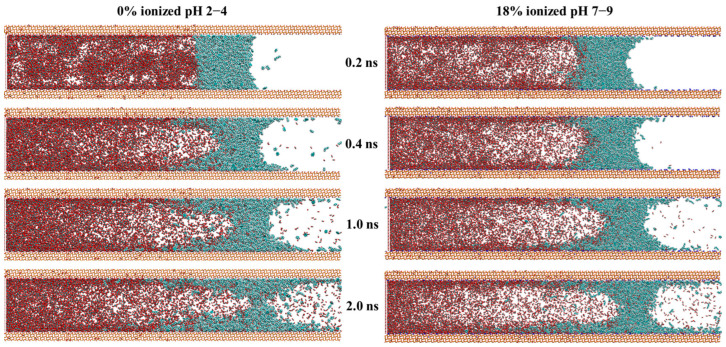
Snapshots of the CO_2_ displacement process within the quartz pore during 0.2–2.0 ns. Red indicates CO_2_ molecules, cyan represents oil-phase molecules, and the top and bottom slab structures simulate the oil reservoir matrix.

The interaction between CO_2_ and crude oil in tight reservoirs, along with its impact on the miscible phase behavior, was characterized by complex mechanisms, although accurate analysis at the nanoscale remained a challenge [33,34]. In this section, the mechanism of the displacement of crude oil in nanopores by CO_2_ is investigated using molecular dynamics methods. The oil phase shown in Figure 5 corresponds to the equilibrated hydrocarbon mixture from Figure 2, including alkanes, cycloalkanes, and aromatic hydrocarbons. However, in Figure 5, all oil components are uniformly colored cyan without further differentiation by molecular type. The dynamic behavior of CO_2_ in displacing crude oil under different pH conditions was visualized in Figure 5. Due to the differing amounts of CO_2_ on the left and right sides of the oil film in the initial configuration, a pressure differential was induced, which resulted in a unidirectional displacement of the oil phase (CO_2_ was present only on the left side of the oil phase, while the right side contained no CO_2_). As shown in Figure 5, as the simulation progressed, the oil phase exhibited overall displacement behavior under both pH conditions, which confirmed the effectiveness of CO_2_ in oil displacement. However, as the simulation advanced, the strong attractive interactions between the crude oil and the quartz surface were found to significantly hinder the efficiency of CO_2_ displacement. Specifically, these strong attractive interactions impeded the interfacial detachment process between the CO_2_ and the oil phase, preventing CO_2_ from rapidly and effectively stripping crude oil from the quartz surface. Consequently, a substantial number of crude oil molecules remained tightly adsorbed on the quartz surface, which reduced the fluidity of the crude oil and the displacement efficiency of the CO_2_. This phenomenon indicated that, when optimizing CO_2_-enhanced oil recovery techniques, not only the interactions between CO_2_ and crude oil but also the influence of reservoir surface adsorption effects need to be thoroughly considered. Additionally, the displacement effect under high-pH conditions was limited by a slower displacement rate. In contrast, the oil phase displacement under low-pH conditions was observed to be more rapid, which suggested that the displacement efficiency of the CO_2_ was significantly enhanced under low-pH conditions. This observation highlighted the pronounced influence of pH on the flow velocity of the oil phase during the CO_2_ displacement process.

**Figure 6:**
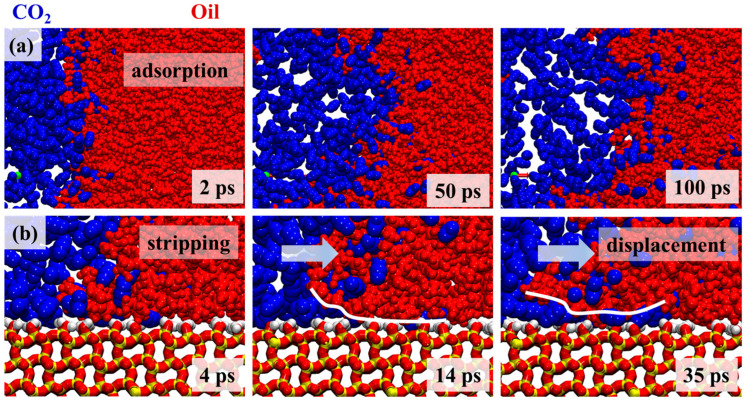
(**a**) Miscible phase behavior of CO_2_ molecules and crude oil. (**b**) Stripping and displacement behavior of CO_2_ molecules on the quartz surface. Blue represents CO_2_ molecules and red represents crude oil molecules.

The miscible and displacement mechanisms of CO_2_ molecules were further investigated in detail. From Figure 5, it can be seen that the initial step of CO_2_ displacement was characterized by the miscible behavior between the CO_2_ molecules and crude oil components, which indicated that CO_2_ miscible displacement could be applied in reservoirs with significant pressure gradients. The initial miscible behavior is visualized in Figure 6. At 2 ps after the simulation began, a clear and stable oil–gas interface with a distinct boundary was observed (Figure 6a). As the simulation progressed, CO_2_ molecules gradually penetrated the oil phase, and miscible phenomena began to emerge. Initially, the CO_2_ molecules were primarily concentrated near the oil–gas interface, making preliminary contact with oil phase molecules. Over time, the CO_2_ molecules were observed to diffuse further into the oil phase interior, engaging in increased interactions with crude oil molecules. This miscible phenomenon suggested that the dissolution and diffusion of CO_2_ in the oil phase progressively increased, which facilitated the displacement and mixing of the oil phase. Following the miscible phase, the CO_2_ displacement behavior was captured in simulation snapshots, as shown in Figure 6b. It was determined from the figure that the overall displacement mechanism could be summarized as an “adsorption-stripping-displacement” process. At the onset of the simulation, CO_2_ molecules were first adsorbed onto the quartz pore surface, forming an adsorption layer. This process is illustrated in Figure 4b, which shows that CO_2_ molecules were significantly aggregated on the quartz pore surface (4 ps), interacting with the pore wall to form a stable adsorption layer. The adsorption phase laid the foundation for the subsequent displacement process. As the simulation advanced, CO_2_ molecules began to diffuse within the crude oil phase and gradually penetrated the gaps within the quartz pores (14 ps). At this stage, interactions between the CO_2_ and crude oil molecules on the quartz surface were observed, which led to the detachment of crude oil molecule edges, which then entered the oil phase. The key to the stripping stage was that crude oil components were successfully detached from the quartz surface through the dissolution and diffusion of CO_2_ molecules. Subsequently, under the driving force of external pressure, the stripped crude oil components were progressively displaced. During this process, the continuous injection of CO_2_ and the applied pressure promoted the movement and mixing of the detached crude oil components within the pore space, and an effective displacement process was thereby achieved. Crude oil molecules were gradually propelled forward under the action of CO_2_, completing the overall displacement process.

**Figure 7:**
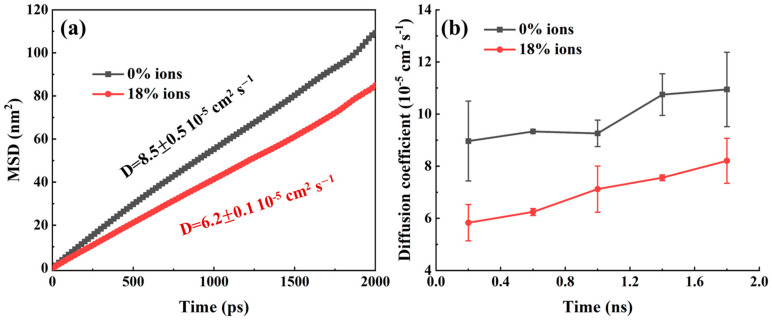
(**a**) Mean square displacement and diffusion coefficient of oil phase molecules during their advancement within the pore. (**b**) Diffusion coefficients of oil phase molecules at different time stages.

The kinetic parameters of oil components during the displacement process under different pH conditions were quantitatively compared to evaluate the influence of pH on CO_2_ flooding. The mean square displacement (MSD) of the oil components throughout the entire displacement process was first calculated, as shown in Figure 7a. It was observed that the oil components exhibited a higher MSD under pH 7–9 conditions, with a diffusion coefficient of 8.5 × 10^−5^ cm^2^ s^−1^, which represents an approximately 37% increase compared to the diffusion coefficient of 6.2 × 10^−5^ cm^2^ s^−1^ at pH 2–4. This indicated that the displacement efficiency of CO_2_ increased with a rising pH. The calculation of time-dependent diffusion coefficients (Figure 7b) further supported this conclusion. This behavior was attributed to the increased negative charge density on the quartz surface under high-pH conditions, which promoted the aggregation of Na+ ions and consequently weakened the adsorption interactions between the oil components and the quartz surface. The weakened adsorption enabled more free movement of the oil molecules within the pore space, which led to enhanced diffusion. In addition, the high pH environment might have altered the viscosity of the pore fluid or the interactions between the fluid and the oil phase, further facilitating the diffusion of oil molecules. These results demonstrated that the pH exerted a significant influence on the dynamic behavior of the oil components, and the higher diffusion coefficients and MSD values under high-pH conditions revealed the potential advantage of CO_2_ flooding under alkaline environments.

## 3. Methodology

In this section, an adsorption model of crude oil components in tight reservoirs is constructed, as shown in Figure 1a, where silica is selected to represent the reservoir solid. The main focus of this study was to investigate the adsorption mechanisms under different pH conditions; therefore, silica models responsive to various pH environments were developed. As illustrated in Figure 1b, pH primarily affects the degree of ionization on the silica surface. Three silica surface models with different ionization degrees were established, corresponding to 0%, 9%, and 18% ionization and representing pH ranges of 2–4, 5–7, and 7–9, respectively [21,22,35]. A certain proportion of alkane, cycloalkane, and aromatic molecules was subsequently placed between the reservoir solids to simulate the adsorption behavior of different crude oil components. The oil phase was modeled using a representative mixture of alkanes, cycloalkanes, and aromatics (heptane: 165, octane: 188, nonane: 170, cycloheptane: 174, cyclohexane: 165, benzene: 107, toluene: 93.) which reflects typical SARA fractions in crude oil [36]. The hydrocarbon molecules were initially placed in a central region between two parallel silica surfaces that formed a nanopore. Care was taken to ensure no initial overlap and to maintain sufficient spacing between molecules to avoid artificial steric effects. The system was then equilibrated under NPT conditions before production runs. Molecular dynamics simulations were conducted under the NPT ensemble for 20 ns. The system was equilibrated under the NPT ensemble, where the number of particles (N), the pressure (P), and the temperature (T) were maintained as constant, which allowed for the realistic simulation of fluid behavior under reservoir conditions. To simplify this study, a nanopore model (Figure 1c) was constructed to approximate the matrix environment of tight reservoirs, while ensuring that the thickness of the pore walls exceeded the cutoff radius of the potential function. The number of CO_2_ molecules introduced into the simulation system was determined based on the target density of CO_2_ at ambient pressure (1 atm) and 358 K, which reflects post-injection diffusion or surface-exposed environments in enhanced oil recovery scenarios [8,37].

Given that the pore size in tight reservoirs typically ranges from 3 to 8 nm, the pore size in this work was set to 7.5 nm, as shown in Figure 1a. A graphene layer was positioned at the starting point on the left side of the simulated lattice to prevent carbon dioxide molecules from crossing the left periodic boundary [38,39]. MD simulations were performed using the GROMACS software 2019 package, employing the General Amber Force Field (GAFF) [40]. The CO_2_ molecules were modeled using the EPM2 model. The simulation temperature was maintained at 358 K using the V-rescale thermostat [41]. The temperature of 358 K (85 °C) was selected to approximate realistic reservoir conditions typically encountered in deep or intermediate-depth tight reservoirs. A cutoff distance of 1.2 nm was applied. The LINCS algorithm was used to constrain bonds involving hydrogen atoms [42], with a time step of 2 fs. Long-range electrostatic interactions were calculated using the particle mesh ewald (PME) method. Trajectory visualization was carried out using the VMD software [43].

## 4. Conclusions

This study was conducted to investigate the adsorption behavior of crude oil on quartz surfaces under varying pH conditions and its impact on the CO_2_ displacement process using molecular dynamics simulations. The main conclusions can be summarized as follows: (1) Crude oil molecules were primarily distributed in the pore bulk phase and on the surface within quartz pores, with the adsorption peak of aromatic hydrocarbons on the quartz surface being significantly higher than that of other components. Moreover, the adsorption of aromatic hydrocarbons was enhanced as the pH increased. (2) Under high-pH conditions, the aggregation of Na+ ions on the quartz surface was found to strengthen cation–π interactions, which further attracted aromatic hydrocarbon molecules and led to the formation of a denser adsorption layer on the quartz surface. (3) The CO_2_ displacement process was characterized as an “adsorption-stripping-displacement” mechanism. An adsorption layer of CO_2_ was initially formed on the quartz surface, which was followed by its penetration into the oil phase, where interactions with crude oil molecules occurred, resulting in the detachment of crude oil components. Ultimately, the detached crude oil components were effectively displaced under the influence of external pressure. (4) The displacement effect under high-pH conditions was limited by a slower displacement rate, whereas the oil phase displacement under low-pH conditions was observed to be more rapid. This was attributed to the tighter packing of aromatic hydrocarbon molecules on the quartz surface under high-pH conditions.

Building on the molecular insights gained in this study, future research should focus on systematically exploring the influence of varying hydrocarbon compositions and more complex oil mixtures on the adsorption and displacement behaviors within nanoporous media. Additionally, experimental validation of the simulation results could be pursued, and alternative strategies for improving displacement techniques, such as the integration of other chemical agents or enhanced CO_2_ injection methods, could be investigated to achieve more efficient reservoir development.

## Data Availability

The original contributions presented in this study are included in the article. Further inquiries can be directed to the corresponding authors.
